# Reactive Oxygen Species (ROS) Generation Is Indispensable for Haustorium Formation of the Root Parasitic Plant *Striga hermonthica*

**DOI:** 10.3389/fpls.2019.00328

**Published:** 2019-03-22

**Authors:** Syogo Wada, Songkui Cui, Satoko Yoshida

**Affiliations:** ^1^Division of Biological Science, Graduate School of Science and Technology, Nara Institute of Science and Technology, Ikoma, Japan; ^2^Division for Research Strategy, Institute for Research Initiatives, Nara Institute of Science and Technology, Ikoma, Japan

**Keywords:** parasitic plant, ROS, peroxidase, haustorium, inhibitor, DMBQ, *Striga*

## Abstract

The parasitic witchweed *Striga hermonthica* causes devastating damage to crops in sub-Saharan Africa, yet the mechanism of its parasitism is not well understood. Parasitic plants form a special organ called a haustorium to obtain water and nutrients from host plants. The haustorium is induced by host-derived small molecules, collectively named haustorium-inducing factors (HIFs). The most active HIF known to date is 2,6-dimethoxy-*p*-benzoquinone (DMBQ), originally isolated from sorghum root extracts. It has been suggested that DMBQ is produced by oxidation of its precursor, syringic acid, and that reactive oxygen species (ROS) and peroxidases are involved in the process. However, the roles of ROS in haustorium formation after HIF recognition remain to be elucidated. Here, we investigated the effects of various inhibitors of ROS and ROS-regulating enzymes on haustorium formation in *S. hermonthica*. Inhibitors of NADPH oxidases and peroxidases inhibited haustorium formation during treatment with DMBQ, syringic acid, and host root extracts, suggesting that ROS production and/or regulation *via* NADPH oxidases and peroxidases are essential for haustorium formation. We observed hydrogen peroxide accumulation in the haustorium upon treatment with various HIFs. Our results suggest that ROS and ROS-regulating enzymes are indispensable in downstream signaling of HIFs for haustorium formation.

## Introduction

*Striga hermonthica*, a noxious parasitic plant belonging to the Orobanchaceae family, infects economically important cereals, such as sorghum, maize, pearl millet, and rice, and causes huge damage to world agriculture, especially in sub-Saharan Africa. The estimated annual yield losses are over one billion USD, negatively affecting the livelihoods of more than 100 million people (Ejeta, 2007). However, the infection mechanism of *S. hermonthica* remains poorly understood and thus effective control methods for this pest have not yet been established.

*S. hermonthica* is an obligate root parasite, depriving the host plant of water and nutrients. *Striga* identifies the presence of potential hosts at the time of its germination by recognition of host-derived strigolactones (Yoshida and Shirasu, 2012). After germination, the *Striga* radicle grows toward host roots and forms a special invasive organ, the haustorium, at the tip of its radical. Haustorium formation is characterized by the swelling of the radicle tip and proliferation of hair cells, haustorial hairs, on the surface. The haustorium functions in host attachment, invasion and nutrient transfer, and its formation is critical for the survival of *Striga* as an obligate parasite (Yoshida et al., 2016). Haustorium formation is initiated after recognition of haustorium-inducing factors (HIFs) derived from host plants. The *Striga* radicle then halts its growth, and cell expansion and division occur at its tip to form a bulge-like structure. At the same time, epidermal cells on the haustorium-forming sites start to proliferate haustorial hairs (O'Malley and Lynn, 2000). When haustoria are induced by HIFs *in vitro*, the bulged haustorium structure is formed within 24 h, and stops further development. Upon infection of a living host, haustoria attach to host tissues and internally invade host roots. Inside host tissues, the parasitic haustoria differentiate to form intrusive cells, some of which further differentiate into xylem cells to connect the host xylem with their own upon entry into the host stele (Yoshida et al., 2016). Overall, haustorium formation involves a cascade of molecular and cellular events controlled in a highly precise spatial-temporal manner.

HIFs are known to be derived from host roots. In 1986, Chang and Lynn identified 2,6-dimethoxy-1,4-benzoquinone (DMBQ) as a HIF from root extracts of sorghum, a *Striga* host plant (Chang and Lynn, 1986). In addition to DMBQ, structurally similar quinones and flavonoids were reported to induce haustoria of *Striga* and the facultative parasitic plants *Triphysaria versicolor* and *Phtheirospermum japonicum* (Albrecht et al., 1999; Cui et al., 2018). Structure-activity relationship studies in *Striga asiatica* found that HIFs fall within a range of certain redox potentials, suggesting that redox potential is critical for HIF activity (Smith et al., 1996). More recently, we have shown that phenolic compounds with one or two methoxy groups at the 3- and 5-positions and an hydroxyl group at the 4-position have activity for haustorium induction in *S. hermonthica* (Cui et al., 2018). Plant cell wall lignin polymers are composed of such phenolic compounds, suggesting that the HIFs may originate from lignin. Alteration of the lignin composition in plants affects haustorium-inducing activity, indicating that HIFs indeed originate from lignin biosynthesis or degradation pathways (Cui et al., 2018). Syringic acid, a phenolic acid potentially produced from the degradation of cell wall lignin, can be oxidized and converted to DMBQ (Kim et al., 1998). Based on this data, Keyes et al. (2001) proposed a model in which host cell wall phenolic acid constituents are oxidized by *Striga*-derived hydrogen peroxide (H_2_O_2_) and peroxidase to produce DMBQ. They showed that reduction of H_2_O_2_ by the H_2_O_2_-scavenging enzyme catalase reduced the haustorium formation rate upon treatment with syringic acid but not with DMBQ (Keyes et al., 2000). Thus, in the model, the reactive oxygen species (ROS) produced by *Striga* are the rate-limiting molecules for the conversion of phenolic acids to DMBQ (Keyes et al., 2000). Furthermore, quinone oxidoreductases (QRs) have been reported to play an important role in haustorium induction of *T. versicolor* and in *P. japonicum* (Bandaranayake et al., 2010; Ishida et al., 2017). Because the QR protein reduces one or two electrons and produces ROS, it was proposed that ROS may also be involved in the haustorium-induction signal (Bandaranayake et al., 2010).

ROS are highly reactive metabolites containing oxygen and are produced in various metabolic processes. Representative ROS are superoxide (${\text{O}}_{2}^{-}$), hydroxyl radical (^·^OH), H_2_O_2_, and singlet oxygen (^1^O_2_). Because of their high reactivity, accumulation of ROS causes oxidative stress and adversely affects most living organisms (Mittler, 2017); however, a moderate amount of ROS is necessary for growth and for signaling induction in response to environmental stress in plants. In *Arabidopsis*, treatment with ROS inhibitors impairs root growth (Dunand et al., 2007). ROS also work as important signals in the immune response to pathogens in plants (Mittler, 2017). Upon pathogen infection, pathogen-associated molecular patterns are recognized by cell surface receptors and activation of calcium channels causes an influx of calcium ions into cells. Subsequent activation of NADPH oxidase by calcium-dependent kinase produces H_2_O_2_, which functions as a signaling molecule (Kadota et al., 2015; Qi et al., 2017). Peroxidases catalyze the production or regulation of ROS, depending on the catalytic activity of each peroxidase isoform, and also contribute to the pathogen response, cell wall lignification and cell elongation (Kim et al., 2010; Das and Roychoudhury, 2014; Shigeto and Tsutsumi, 2016). Despite the importance of ROS regulation in plant growth and development, the roles of ROS in host-parasitic plant interaction remain poorly understood.

In this study, we aimed to investigate the roles of ROS and related enzymes in haustorium-induction signaling in *S. hermonthica*. Our pharmacological study found that NADPH oxidases and peroxidases have important roles in haustorium formation before and after perception of HIF signals.

## Materials and Methods

### Plant Materials

*Striga hermonthica* seeds were kindly provided by Prof. A. G Babiker (National Research Center, Khartoum, Sudan). Approximately 50–100 mg of *Striga* seeds were placed into 1.5 ml plastic tubes and sterilized with 20% commercial bleach solution (Kao Ltd., Japan; final concentration of sodium hypochlorite was about 0.6%). The seeds in the bleach solution were mixed by vortexing, left to stand for several seconds to precipitate, and the supernatant was removed. This sterilization procedure was repeated 10 times. The *Striga* seeds were then rinsed 10 times with sterile water and transferred to 9 cm petri dishes with moistened glass fiber filter paper (GE Healthcare UK Ltd., Little Chalfont, UK). For preconditioning, the petri dish was sealed using surgical tape and incubated at 25°C for 7 d in the dark. Preconditioned *Striga* seeds were treated with 10 nM strigol (Hirayama and Mori, 1999) at 25°C for 1 d in the dark, and then subjected to haustorium-inducing factor treatments.

For preparation of rice root extracts, rice (*Oryza sativa* subsp. *japonica* cv. Koshihikari and cv. Nipponbare) seeds were briefly washed with 70% ethanol, sterilized with 10% commercial bleach solution (Kao Ltd., Japan; final concentration of sodium hypochlorite was about 0.3%) for 30 min and washed at least three times using sterilized water. For germination, the sterilized seeds were transferred to 9 cm petri dishes with moistened filter paper (Advantec Co Ltd., Tokyo, Japan) and incubated at 25°C for 7 d under 16 h light (35 μmol/sm^2^)/8 h dark conditions. The rice roots were collected, ground under liquid nitrogen to a fine powder and suspended in sterilized water to a concentration of 5% (w/v). The rice extract solution was filter-sterilized and mixed with agar medium to the final concentration indicated.

### Chemicals and Reagents

Chemicals used in this study were obtained from the following suppliers; DMBQ (Sigma-Aldrich, Osaka, Japan), syringic acid (Sigma-Aldrich), salicylhydroxamic acid (SHAM, Tokyo Chemical Industry Co., Ltd., Tokyo, Japan), potassium iodide (Wako Pure Chemical Industries, Ltd., Osaka, Japan), potassium benzoate (Wako Pure Chemical Industries, Ltd.), L-ascorbic acid (Wako Pure Chemical Industries, Ltd.), diphenyleneiodunium (DPI, Funakoshi Frontiers Life Science, Tokyo, Japan), umbelliferone (Tokyo Chemical Industry Co., Ltd.), 6,7-dihydroxycoumarin (esculetin, Sigma-Aldrich), phenylarsine oxide (PAO, Sigma-Aldrich), and sodium diethyldithiocarbamate trihydrate (DDC, Sigma-Aldrich). Each chemical was dissolved in DMSO or water at a stock concentration of 10 mM and stored at −20°C until use (Supplementary Table S1).

Enzymes used in this study were obtained from the indicated supplier; superoxide dismutase (SOD, Nacalai Tesque, Inc., Kyoto, Japan), catalase (Wako Pure Chemical Industries, Ltd.), and horse radish peroxidase (HRP, Wako Pure Chemical Industries, Ltd.). SOD, HRP, and catalase were dissolved in phosphate buffer (pH 7.0) at a stock concentration of 10^4^, 10^4^, and 10^5^ U/ml, respectively, and stored at −20°C until use. H_2_O_2_ was obtained from Nacalai Tesque.

### Visualization of ROS

Intracellular localization of H_2_O_2_ was visualized using carboxy-H2DFFDA (Thermo Fisher). Strigol-treated *Striga* seeds were transferred to plastic 96-well plates with 20–30 seeds per well. One hundred microliter of DMBQ (Sigma-Aldrich) or syringic acid (Sigma-Aldrich) were added to each well at a final concentration of 10 μM and placed at 25°C in the dark. For the control treatment, an equal amount of water was used. After 24 h, the solution in each well was removed and the seedlings were stained with 100 μl of 20 μM carboxy-H2DFFDA reagent for 30 min. The stained seedlings were rinsed with sterile water a few times and observed under a confocal microscope (Leica TCS SP5), using 460 and 508 nm as excitation and emission wavelengths, respectively. For time-lapse photographs, germinated *Striga* seedlings were mounted on a glass-bottom dish (Matsunami Glass Ind., Ltd., Osaka, Japan) with 10 μM DMBQ and 20 μM carboxy-H2DFFDA, and immediately observed under a Leica TCS SP5 microscope. Time-lapse photos were taken every 30 min for 24 h with 460 and 508 nm excitation and fluorescence wavelengths, respectively.

For ${\text{O}}_{2}^{-}$ visualization, haustoria were induced by 10 μM DMBQ or syringic acid for 24 h and the seedlings were stained with 0.5 mg/ml Nitroblue tetrazolium chloride (NBT: Roche) for 30 min. The stained seedlings were washed with sterile water a few times and observed under light microscopy.

For visualization of NO^−^ and ^·^OH, *Striga* haustoria induced by 10 μM DMBQ or syringic acid for 24 h were stained with 10 μM diaminoflurescein-2-diacetate (DAF-2 DA, Goryo Chemical, Inc., Sapporo, Japan) or 10 μM aminophenyl fluorescein (APF, Goryo Chemical, Inc.), respectively, for 30 min, and were then washed with sterile water a few times. The *Striga* seedlings were observed under a confocal microscope (Leica TCS SP5), using 460 and 508 nm as excitation and fluorescence wavelengths, respectively.

### Treatment With ROS Inhibitors and ROS-Related Enzymes

Strigol-treated seeds were transferred to 96-well plates with 20–30 seeds per well. Ten micrometer DMBQ (Sigma-Aldrich) or syringic acid (Sigma-Aldrich), or 0.5% rice root extracts, were added to the germinated *Striga* seeds together with various ROS inhibitors or ROS-related enzymes. The plates were sealed using surgical tape and kept at 25°C in the dark for 24 h. Haustorium formation was observed under a stereo microscope (Zeiss Stemi-2000) and the haustorium formation rate (expressed as a percentage) was calculated as the number of plants that formed an haustorium divided by the total number of plants used in the experiment. At least three biological replicates were performed for each experiment. Statistically significant differences between samples were detected by either a Student's *t*-test or a Tukey HSD (honest significant difference) test using Excel or R, respectively.

## Results

### Effects of ROS Scavengers on Haustorium Induction

**Figure 1:**
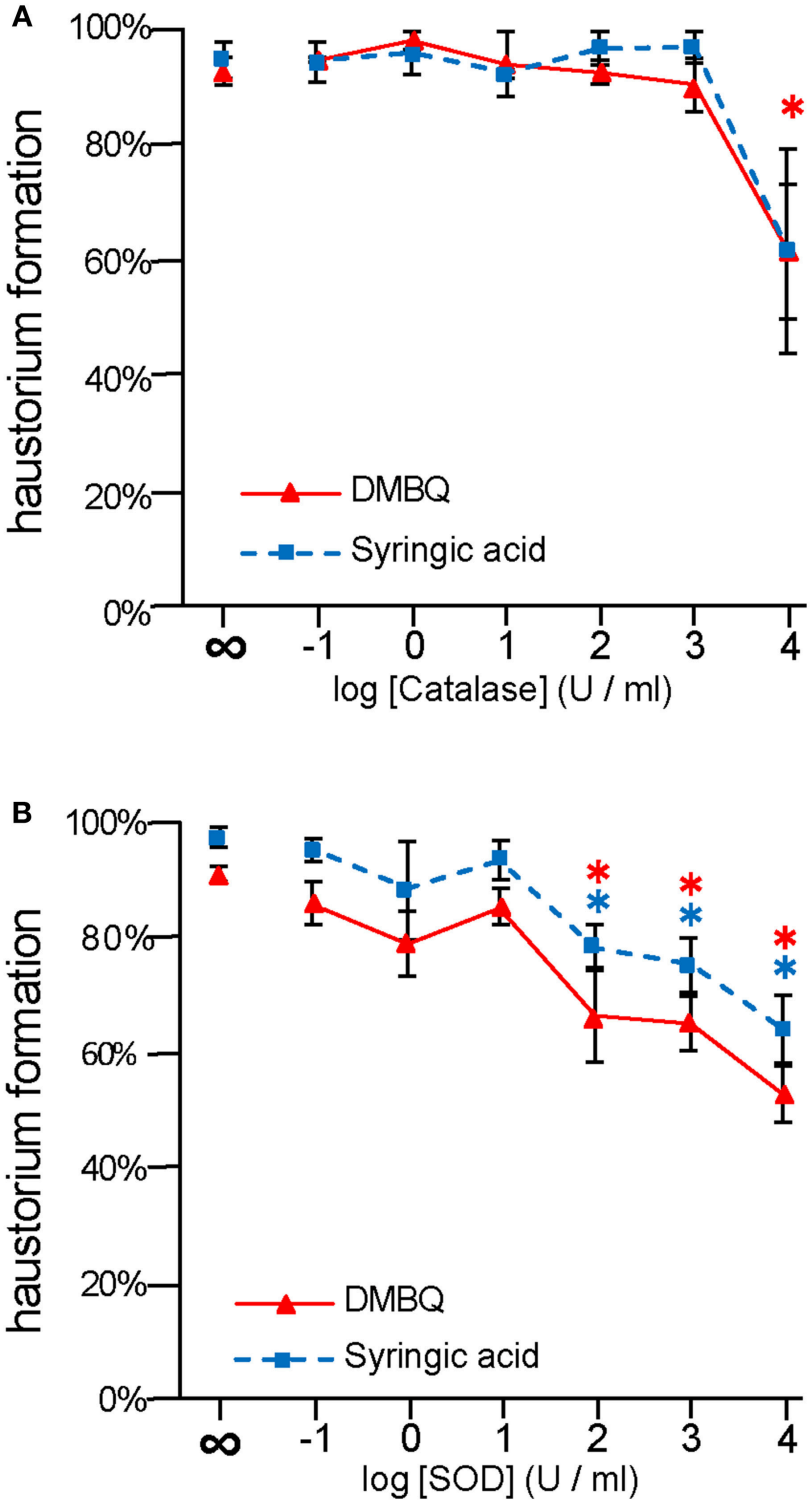
Effects of external application of ROS-scavenging enzymes on haustorium formation. *S. hermonthica* seedlings were exposed to DMBQ or syringic acid with the ROS-scavenging enzymes, catalase **(A)** or SOD **(B)**. Ten micrometer of DMBQ **(A,B)** and 100 μM **(A)** or 10 μM **(B)** of syringic acid were used in each experiment to allow comparison with a previous report on catalase (Keyes et al., 2000). After 24 h exposure, haustorium formation rates were evaluated. Experiments were performed at least twice independently with similar results. Red triangles and blue squares indicate treatment with DMBQ and syringic acid, respectively, and error bars indicate standard error (SE: *n* = 3). ∞ indicates without enzyme control. Asterisks indicate statistically significant differences between samples with and without the indicated enzymes as detected by a Student's *t*-test (*P < 0.05, red asterisk, DMBQ; blue asterisk, syringic acid).

To investigate the importance of ROS-scavenging enzymes on haustorium induction, we performed haustorium induction assays with catalase and SOD, which scavenge H_2_O_2_ and ${\text{O}}_{2}^{-}$, respectively. *Striga* seedlings were exposed to the HIFs, DMBQ, and syringic acid, in the presence of these enzymes for 24 h, and the haustorium formation rate was calculated. Without the addition of enzyme, the haustorium formation rate was almost over 90% with either 10 μM DMBQ or syringic acid. Upon catalase treatment at 10^4^ U/ml, the haustorium formation rate was reduced to 60% in either the DMBQ or syringic acid treatments (Figure 1A). These results indicate that the ROS-scavenger catalase affected haustorium formation in a similar way when induced by either DMBQ or syringic acid.

The haustorium formation rate in the presence of DMBQ or syringic acid was reduced to 60 and 70% at 10^3^ U/ml SOD, respectively. With the addition of 10^4^ U/ml SOD it was reduced to 50% in the presence of DMBQ and 30% in the presence of syringic acid (Figure 1B). These results suggest that removal of ${\text{O}}_{2}^{-}$ from the solution by SOD interfered with haustorium formation. Although there were slight differences in the inhibitory effect of SOD between DMBQ and syringic acid, it appears that ${\text{O}}_{2}^{-}$ was necessary for haustorium formation when induced by either HIFs.

### Localization of ROS During Haustorium Formation in *S. hermonthica*

**Figure 2:**
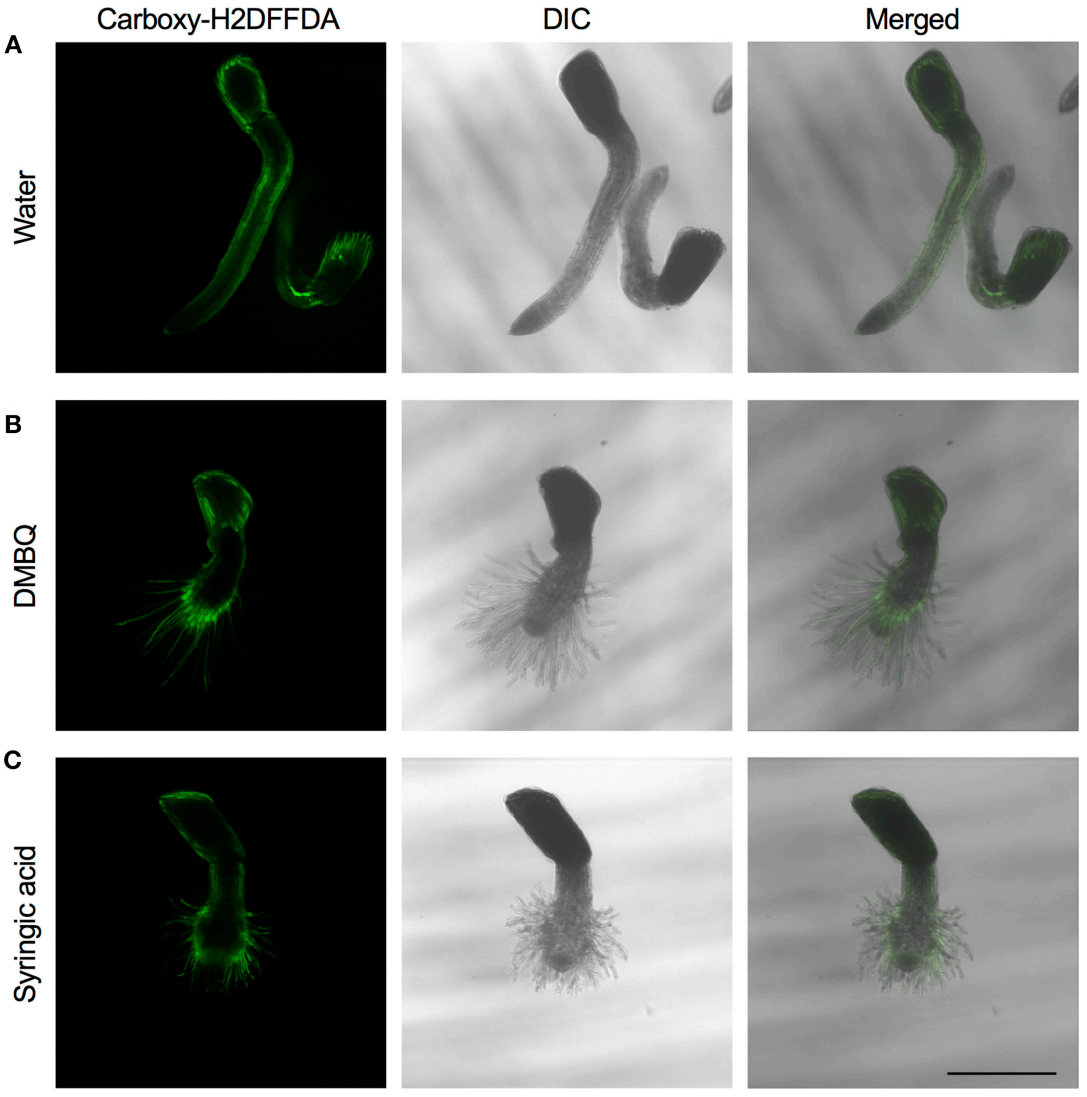
Visualization of H_2_O_2_ accumulation in haustoria of *S. hermonthica. S. hermonthica* seedlings were untreated **(A)** or treated with 10 μM DMBQ **(B)** or 10 μM syringic acid **(C)** for 24 h and accumulation of H_2_O_2_ was visualized by carboxy-H2DFFDA staining. Fluorescence images (left panels), DIC images (middle panels), and overlay images (right panels) are shown. Scale bar indicates 500 μm. The experiments were repeated at least three times and representative images are shown.

**Figure 3:**
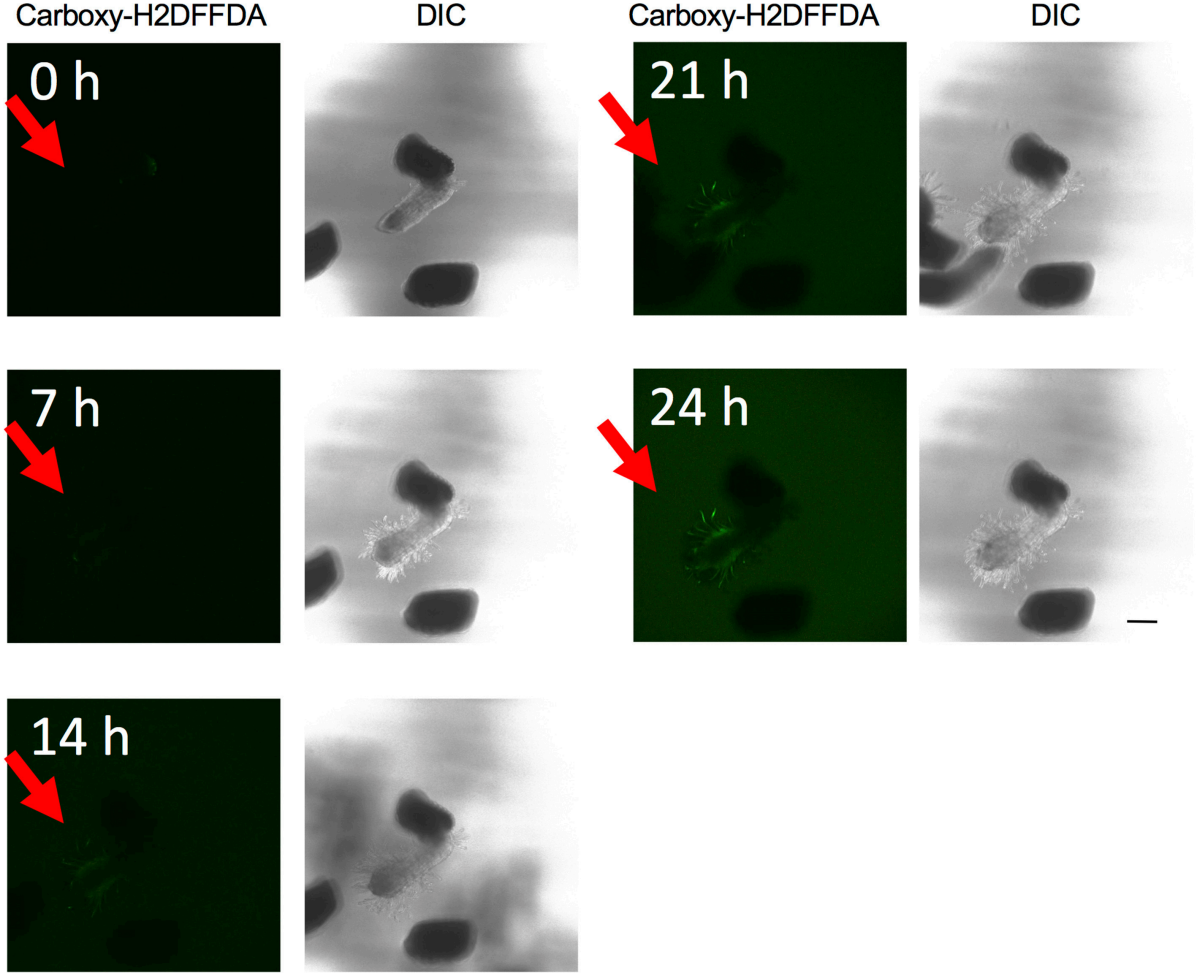
Time course visualization of H_2_O_2_ accumulation. H_2_O_2_ accumulation was observed by carboxy-H2DFFDA staining (green) at 0, 7, 14, 21, and 24 h after haustorium induction by DMBQ. Each time point is composed of a fluorescence image (left) and a DIC image (right). Each image was derived from the same laser power and exposure time. Red arrows indicate *S. hermonthica* radicles. The scale bar indicates 200 μm.

The above experiments indicate that the presence of ROS is important for haustorium induction. To test whether such ROS accumulate in the parasite tissues, we visualized H_2_O_2_ and ${\text{O}}_{2}^{-}$ upon haustorium induction. We used carboxy-H2DFFDA, which is activated by endogenous esterase in presence of H_2_O_2_ to emit a green fluorescence, for detecting H_2_O_2_ accumulation in living cells. Although this probe can also react with other ROS, it has been widely used in plants to monitor real-time H_2_O_2_ production in plants due to its high sensitivity (Swanson et al., 2011). *Striga* haustoria were induced by DMBQ or syringic acid, stained with carboxy-H2DFFDA, and observed under fluorescence confocal microscopy. In the control, water-treated *Striga* seedlings, a moderate H_2_O_2_ signal was detected at the maturation zone of roots and no detectable signal was observed at the root tips (Figure 2A). In contrast to this, haustorium-forming *Striga*, which were treated with DMBQ or syringic acid, strongly accumulated H_2_O_2_ in the haustorial hairs (Figures 2B,C). Next, we observed a time course of H_2_O_2_ accumulation during haustorium formation. The swelling of the radicle tip and proliferation of haustorial hairs were observed approximately 7 h after DMBQ treatment, while at this time point, no significant florescence signal indicating H_2_O_2_ accumulation was detected except a very weak signal at the tip of emerging haustorium. The green fluorescence started to appear in haustorial hairs at 14 h after DMBQ treatment and ultimately covered the whole haustorium surface at 24 h (Figure 3 and Supplementary Movies S1A,B). These results indicated that accumulation of H_2_O_2_ did not precede haustorium formation and was accompanied by morphological changes in the *Striga* radicles.

**Figure 4:**
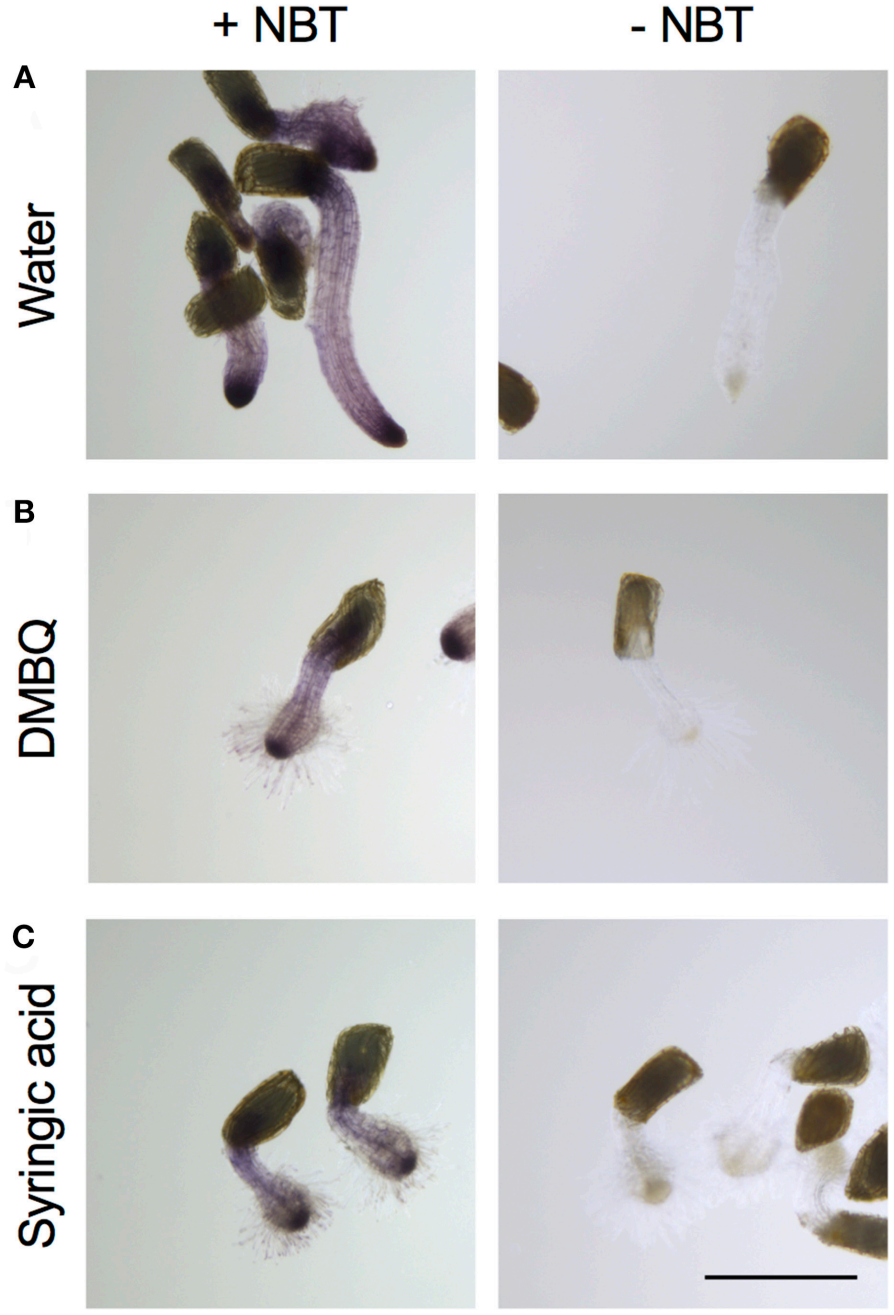
Visualization of ${\text{O}}_{2}^{-}$ accumulation in haustoria of *S. hermonthica. S. hermonthica* seedlings were untreated **(A)** or treated with 10 μM DMBQ **(B)** or 10 μM syringic acid **(C)** for 24 h and stained with NBT to visualize ${\text{O}}_{2}^{-}$. NBT-stained seedlings (left panels) and no-staining control (right panels) are shown. Scale bar indicates 500 μm. The experiments were repeated at least three times and representative images are shown.

For detection of ${\text{O}}_{2}^{-}$, *Striga* haustoria were stained with NBT. In the roots of water-treated *Striga* seedlings, low levels of NBT staining were observed throughout the plant, with strong staining at the root tip (Figure 4A). After haustorium induction by DMBQ or syringic acid, the haustorium tip region was strongly stained by NBT, while the other plant parts including haustorial hairs showed moderate staining (Figures 4B,C). These results suggested that, despite major morphological changes after haustorium induction, ${\text{O}}_{2}^{-}$ was mostly localized at the tips of radicles or haustoria.

We also investigated the localization of NO^−^ and ^·^OH, using DAF-2 DA and APF, respectively. Although NO^−^ signals were observed at a slightly higher level in haustoria than in control roots, these ROS signals were very faint in both the radial tip and the haustorium under our experimental conditions (Supplementary Figure S1). To make conclusions about the accumulation of NO- and ^·^OH during haustorium formation, a more sensitive assay system is required.

### Effects of ROS Inhibitors on Haustorium Induction in *S. hermonthica*

**Figure 5:**
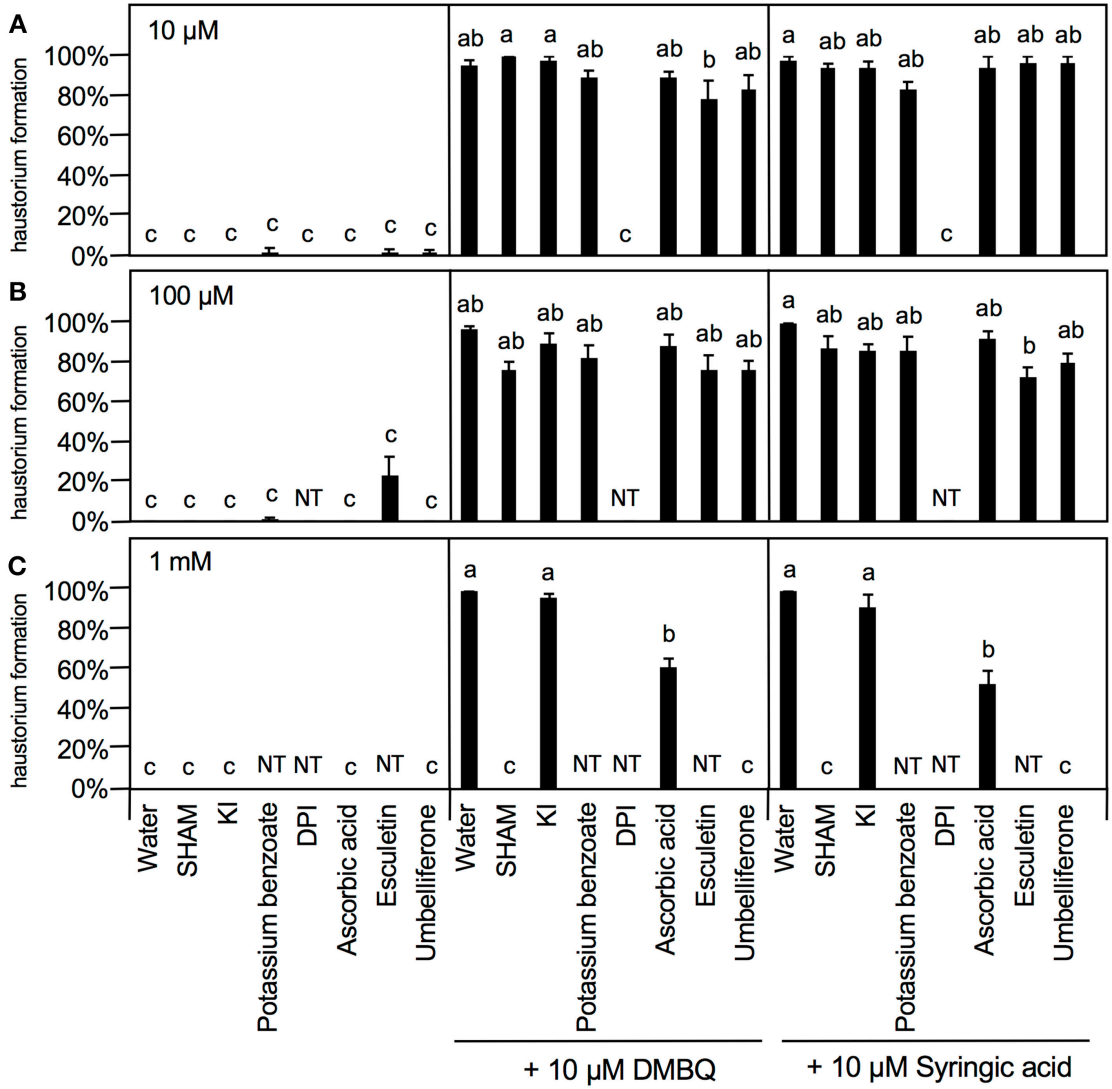
Effects of ROS or ROS-regulating enzyme inhibitors on haustorium formation. Haustorium formation rates were calculated after 24 h treatment with 10 μM **(A)**, 100 μM **(B)**, 1 mM **(C)** of the inhibitors indicated, in the presence of 10 μM DMBQ or syringic acid. Error bars indicate SE (*n* = 3), and NT, not tested. Different lower-case letters represent significant differences as determined by a Tukey HSD test (*p* < 0.05).

To further investigate the involvement of ROS in haustorium induction, various ROS scavengers and inhibitors targeting ROS-regulating enzymes were tested during HIF treatments. We used seven inhibitors or modulators with different efficacies; SHAM (a peroxidase inhibitor), potassium iodide (KI) and potassium benzoate (scavengers of H_2_O_2_), DPI and PAO [NADPH oxidase inhibitors; (Liszkay et al., 2004)], DDC (an inhibitor of SOD), L-ascorbic acid [an oxygen radical scavenger; (Chen et al., 2013)] and umbelliferone and 6,7-dihydroxycoumarin (esculetin) [peroxidase modulators; (Dunand et al., 2007)]. Germinated *Striga* seedlings were exposed to various concentrations of each ROS or enzyme inhibitor together with HIFs for 24 h and the haustorium formation rate was calculated. The inhibitors themselves did not induce haustorium except esculetin, which showed a slight haustorium-inducing activity at 100 μM (Figure 5).

**Figure 6:**
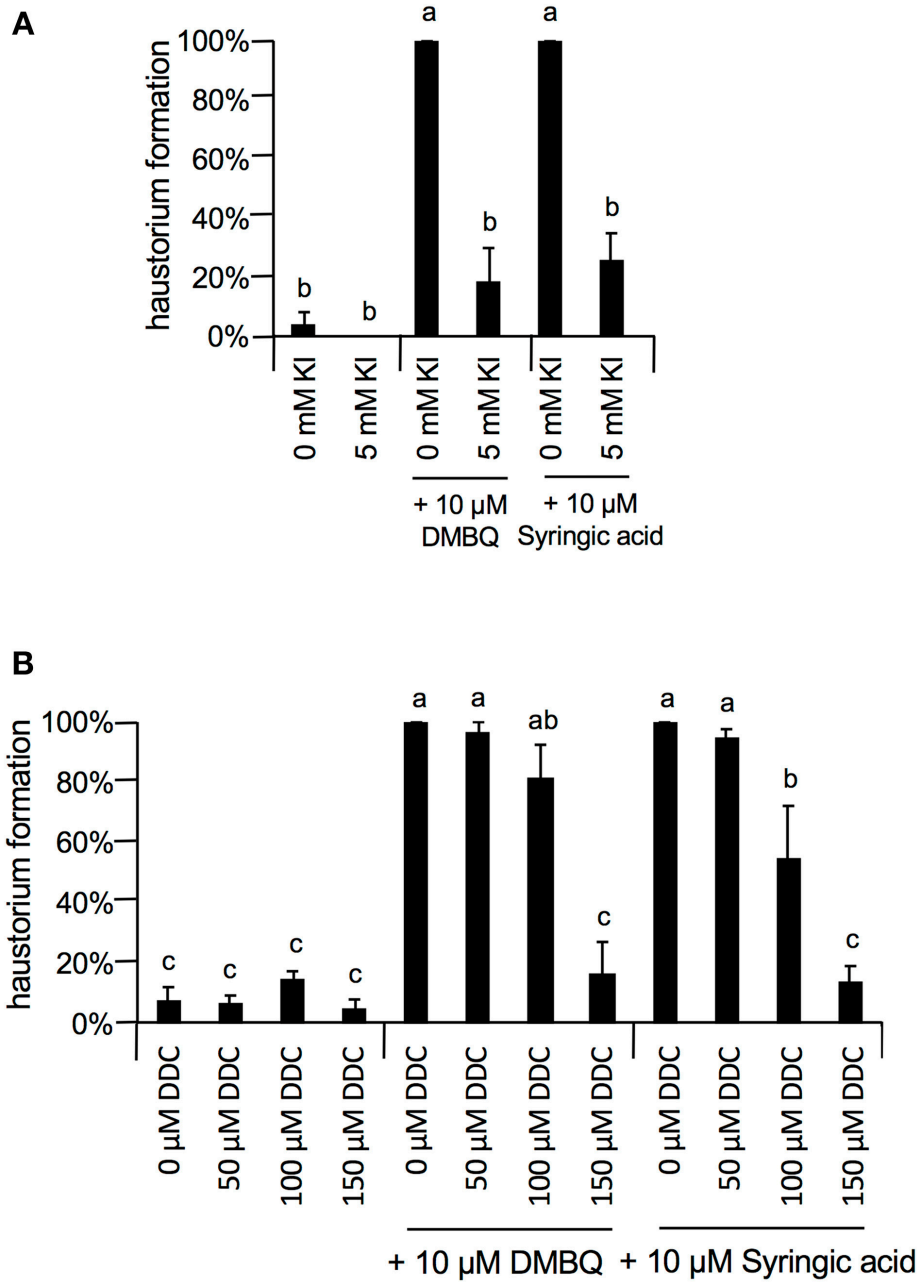
Effects of Kl and DDC on haustorium formation. **(A)** Effect of 5 mM KI on haustorium induction by DMBQ or syringic acid. **(B)** Concentration-dependent inhibition of DDC on haustorium formation induced by DMBQ or syringic acid. Error bars indicate SE (*n* = 3), and NT, not tested. Different lower-case letters represent significant differences as determined by a Tukey HSD test (*p* < 0.05).

At 10 μM, none of the ROS inhibitors displayed any significant inhibitory effects on haustorium formation, except for DPI, which almost entirely blocked haustorium formation (Figure 5A). At 100 μM, esculetin alone significantly reduced haustorium formation by 20% with 10 μM syringic acid treatment (Figure 5B). At 1 mM, SHAM and umbelliferone completely inhibited haustorium formation induced by either DMBQ or syringic acid (Figure 5C, Supplementary Figure S2). Furthermore, 1 mM ascorbic acid reduced the haustorium formation rate to about 60% in both DMBQ and syringic acid treatments (Figure 5C and Supplementary Figure S2). A high concentration (1 mM) of esculetin and potassium benzoate was not tested, because 1 mM esculetin strongly inhibited root tip growth and turned the root tip brown (Supplementary Figure S2), and potassium benzoate was difficult to solubilize at this concentration. The concentration-dependent effects of SHAM were further analyzed. 250 and 500 μM SHAM decreased the haustorium formation rate to about 20 and 0%, respectively (Supplementary Figure S3A). Because KI, a H_2_O_2_ scavenger, was not effective at a concentration of 1 mM, we tested higher concentrations. Treatment with 5 mM KI had almost no effect on root growth of *Striga* seedlings without HIFs (Supplementary Figure S2) and decreased the haustorium formation rate to approximately 20% in both DMBQ and syringic acid treatments (Figure 6A). In addition, we tested PAO as another NADPH oxidase inhibitor. PAO inhibited haustorium formation at 10 μM (Supplementary Figure S3B). These results indicate that NADPH oxidase inhibitors, peroxidase inhibitors and H_2_O_2_ scavengers effectively inhibited haustorium formation in *S. hermonthica*. Because NADPH oxidase produces ${\text{O}}_{2}^{-}$ that is converted to H_2_O_2_ by SOD, we examined the effects of the SOD inhibitor, DDC, on haustorium induction. DDC treatment at 150 μM decreased the haustorium formation rate to 10% (Figure 6B), suggesting that H_2_O_2_ has a pivotal role in haustorium formation in *S. hermonthica*.

The effects of ROS inhibitors on haustorium formation were also examined for rice root extract-induced haustoria, using extracts from the cultivars Nipponbare and Koshihikari. Nipponbare is a *Striga*-tolerant cultivar, while Koshihikari is susceptible (Gurney et al., 2006; Yoshida and Shirasu, 2009). The major HIFs produced by rice root extracts have not been identified to date and it is possible that compounds other than DMBQ or syringic acid are responsible for haustorium induction in this plant. At a concentration of 0.5%, the extracts of both cultivars induced haustoria by over 90% (Supplementary Figure S4). Like the results from haustorium induction by DMBQ or syringic acid, haustorium formation was inhibited by 10 μM DPI, 1 mM SHAM, and 1 mM umbelliferone and partially inhibited by 1 mM ascorbic acid (Supplementary Figure S4). The effects of ascorbic acid were slightly but significantly different between susceptible and resistant cultivars, which may reflect the difference in HIFs produced by the extracts of these two cultivars. Overall, the effects of the inhibitors were similar in both the chemical-induced and root extract-induced haustoria, indicating that haustorium induction by rice root extracts occurred in a similar manner to the induction by DMBQ and syringic acid, at least in regards to ROS function.

**Figure 7:**
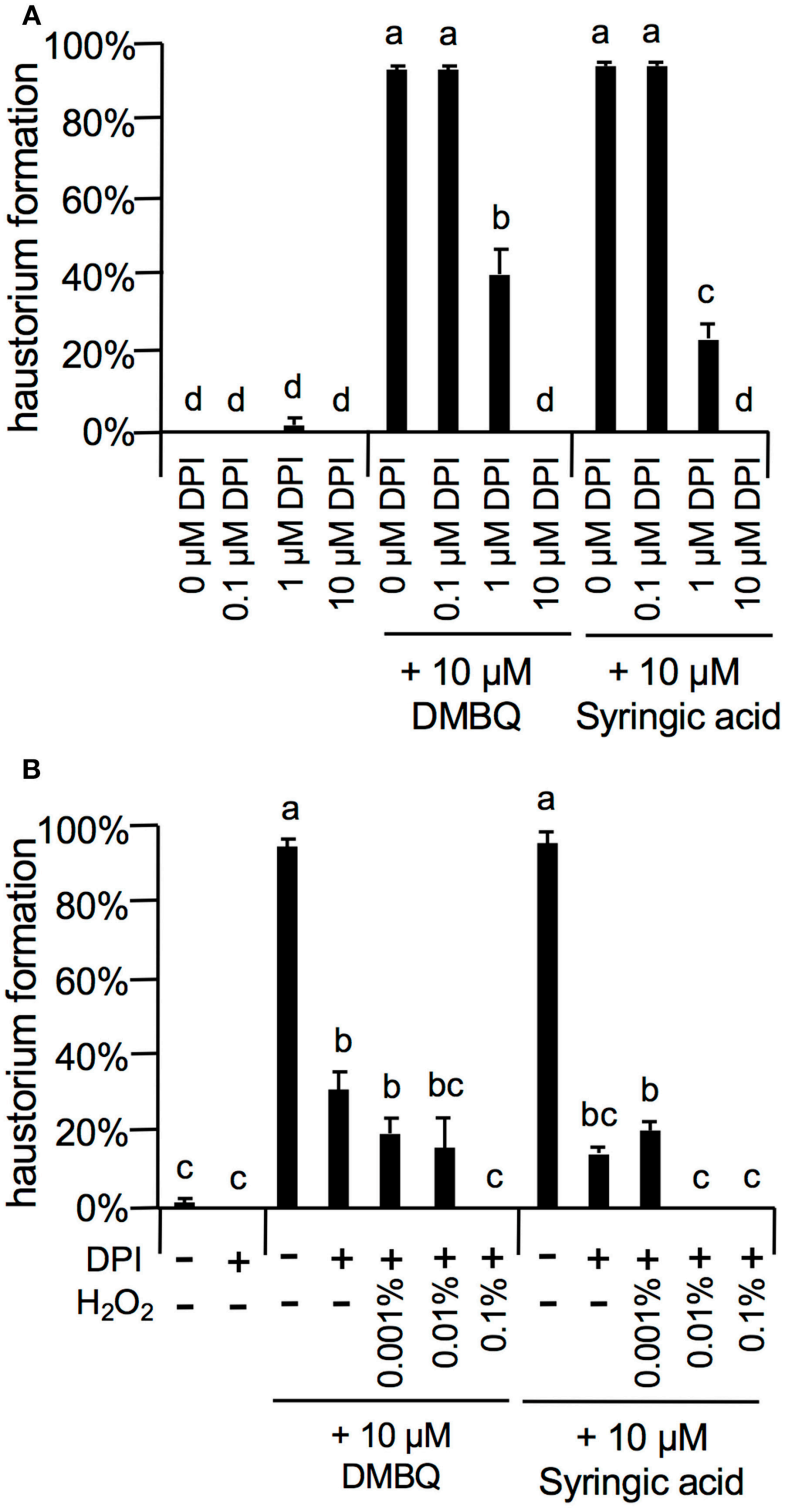
Effects of DPI and external H_2_O_2_ on haustorium formation. **(A)** Haustorium formation rates upon treatment with lower concentrations of DPI in the presence of 10 μM DMBQ or syringic acid. **(B)** Effects of external H_2_O_2_ on haustorium formation rates treated with 1 μM DPI in the presence of 10 μM DMBQ or syringic acid. Error bars indicate SE (*n* = 3). NT, not tested. Different lower-case letters represent significant differences as determined by a Tukey HSD test (*p* < 0.05).

As the strongest inhibition was observed with 10 μM DPI (Figure 7), we investigated the concentration-dependent inhibition of haustorium formation by this NADPH oxidase inhibitor. The inhibitory effects of DPI were confirmed at 1 μM with a reduction of 60 and 80% in DMBQ and syringic acid treatment, respectively (Figure 7A). Inhibition was not detected at 0.1 μM (Figure 7A). Because DPI is an NADPH oxidase inhibitor and NADPH oxidase is involved in ${\text{O}}_{2}^{-}$ and subsequent H_2_O_2_ production, we investigated whether inhibition of haustorium formation by DPI could be restored by exogenous H_2_O_2_ application. Application of exogenous H_2_O_2_ with DPI did not restore the rate of haustorium formation, either induced by DMBQ or syringic acid (Figure 7B). These results indicate that *in planta* H_2_O_2_ generation by NADPH oxidases is crucial for haustorium formation.

### Effects of Exogenous Peroxidase on Haustorium Formation

Previous studies suggest that peroxidases, together with H_2_O_2_, function in catalyzing the oxidation reaction that converts syringic acid to DMBQ (Kim et al., 1998; Keyes et al., 2000). However, our inhibition experiments above indicated that peroxidases may also be involved in haustorium formation by DMBQ. To further test the effect of peroxidase, we applied an exogenous peroxidase and observed its effect on haustorium formation by DMBQ and syringic acid.

**Figure 8:**
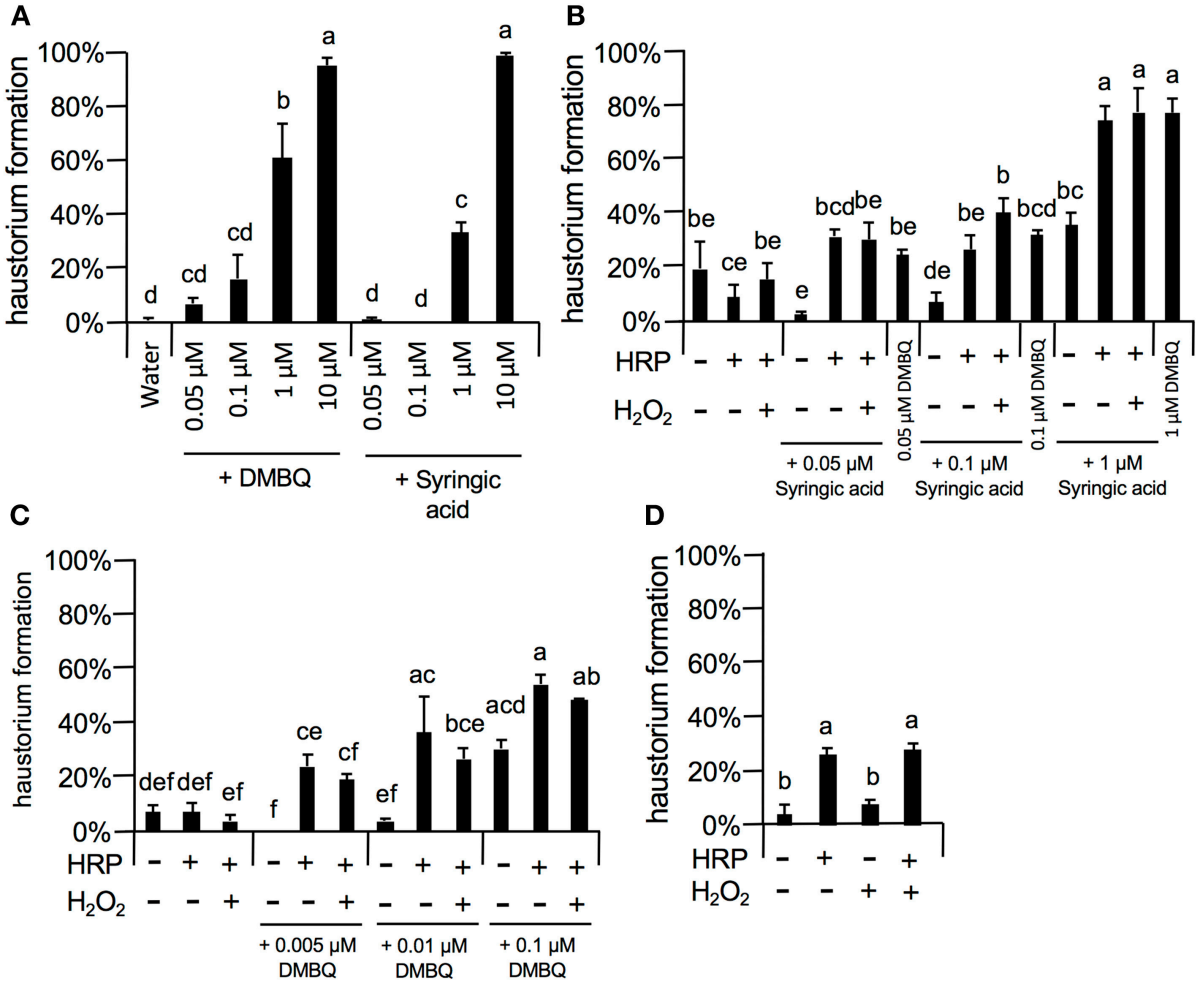
Effects of external peroxidase on haustorium formation by DMBQ and syringic acid. **(A)** Concentration-dependent haustorium formation rates of DMBQ and syringic acid treatments. *S. hermonthica* seedlings were exposed to 0.05, 0.1, 1, and 10 μM of DMBQ or syringic acid, and haustorium formation rates were calculated after 24 h. **(B,C)** *Striga* seedlings were exposed to a combination of 0.01 U/μl HRP and 0.001% H_2_O_2_ in the presence of various concentrations of syringic acid **(B)** or DMBQ **(C)** and haustorium formation rates were calculated after 24 h. **(D)** *S. hermonthica* seedlings were exposed to a high concentration of HRP at 0.1 U/μl and 0.001% H_2_O_2_, and haustorium formation rates were calculated after 24 h. Error bars indicate SE (*n* = 3). NT, not tested. Different lower-case letters represent significant differences as detected by a Tukey HSD test (*p* < 0.05).

As 10 μM DMBQ and syringic acid showed almost saturated haustorium induction rates (over 90%, Figures 5–8), lower concentrations of these molecules were first tested to compare the activity of DMBQ and syringic acid. One, 0.1, and 0.05 μM DMBQ treatment resulted in an approximately 60, 20, and 10% haustorium formation rate, respectively. On the other hand, 1 μM syringic acid induced haustoria at a rate of 40%, and almost no haustorium-induction activity was observed at concentrations of 0.1 μM or less (Figure 8A). These results suggest that DMBQ has a higher haustorium-induction activity than syringic acid in *S. hermonthica*.

Next, to assess the haustorium formation-promoting effects of peroxidase, HRP was applied with DMBQ or syringic acid (Figures 8B,C). When HRP (10 U/ml HRP) was applied together with syringic acid at various concentrations, the haustorium formation rate was significantly increased to similar levels of that induced by DMBQ. For example, application of HRP with syringic acid (0.1 μM) increased the haustorium formation rate to 30%, similar to the rate induced by 0.1 μM DMBQ alone. These results support the proposed model where syringic acid is converted to DMBQ by *Striga* peroxidase (Keyes et al., 2000), at least as measured by haustorium-induction activity. Interestingly, HRP also increased haustorium formation rates with DMBQ (Figure 8C), suggesting that peroxidase has a direct effect on DMBQ or on *Striga*'s perception of HIFs. In contrast to HRP, the addition of H_2_O_2_, the co-substrate of peroxidase, did not enhance haustorium formation, possibly due to the presence of H_2_O_2_ from *Striga* (Figures 8B,C). Surprisingly, higher concentrations of HRP (10^2^ U/ml) in the absence of HIFs also induced haustorium formation at up to 20% (Figure 8D), whereas H_2_O_2_ alone did not promote haustorium induction. These results suggest that HRP itself can enhance the haustorium formation rate of *S. hermonthica* at high concentrations.

## Discussion

### The Role of ROS in Haustorium Formation

**Figure 9:**
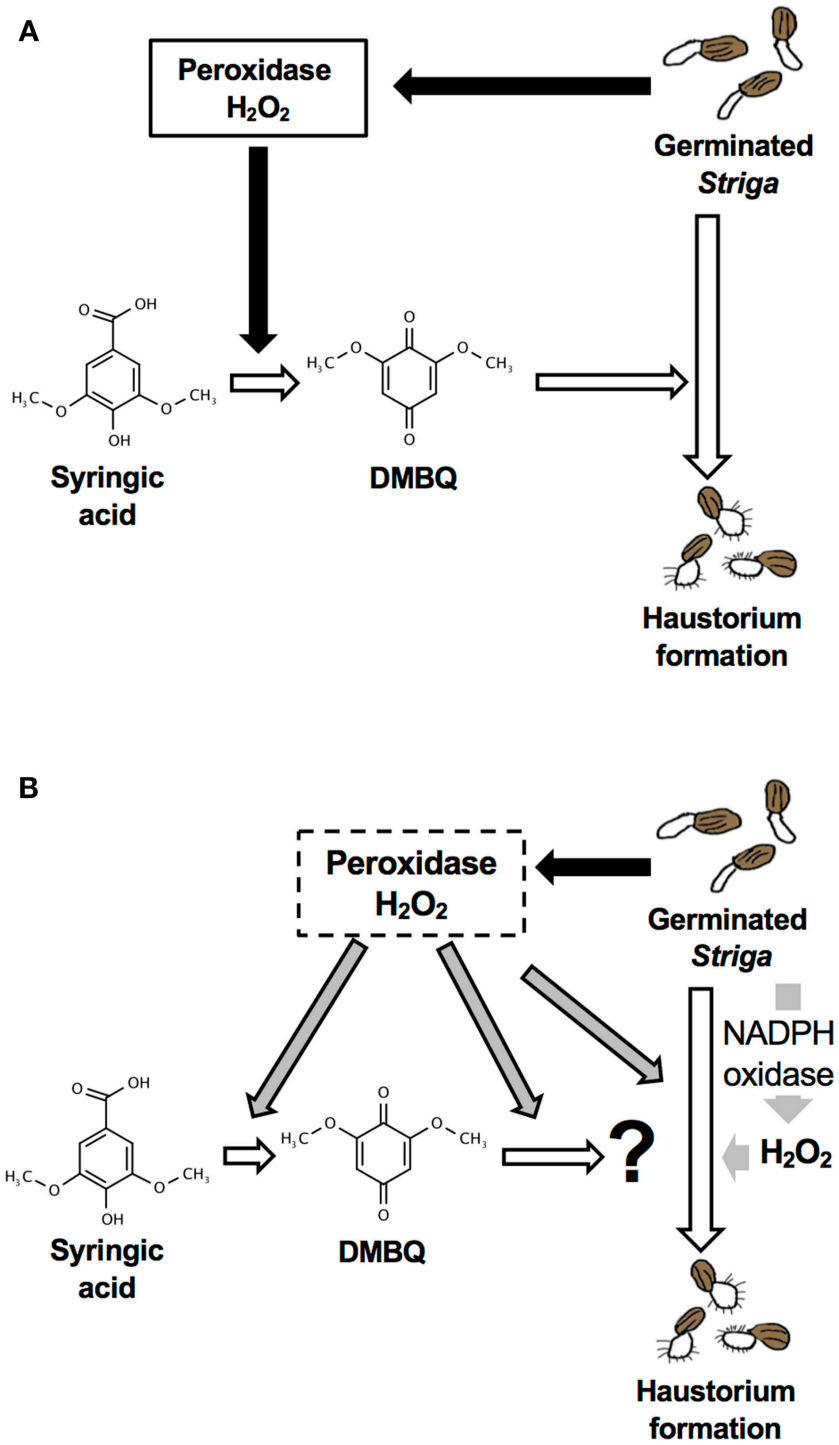
Models for haustorium induction in *S. hermonthica*. **(A)** A model from previous studies [modified from (Keyes et al., 2001)]. DMBQ is produced from syringic acid by *Striga*-derived peroxidase and H_2_O_2_. DMBQ acts as a signal for *Striga* seedlings to form a haustorium. **(B)** A model suggested by this study. *Striga*-derived peroxidase can convert syringic acid to DMBQ, which may be further converted to a compound with higher haustorium-inducing activity. In addition, peroxidase enhances haustorium formation independently from HIFs. NADPH oxidase and H_2_O_2_ production have a pivotal role in haustorium formation in *Striga*.

Although DMBQ was identified as a HIF from sorghum root extracts (Chang and Lynn, 1986), the mode of action of HIFs is poorly understood. Keyes et al. (2001) reported that the haustorium formation rate in *S. asiatica* was reduced by catalase treatment when haustoria were induced by syringic acid but not by DMBQ, and concluded that H_2_O_2_ is important for the conversion of syringic acid to DMBQ during haustorium formation [Figure 9A; (Keyes et al., 2001)]. However, our results show that catalase (a H_2_O_2_ scavenger) and SOD (an ${\text{O}}_{2}^{-}$ scavenger) decrease the rate of haustorium formation even when DMBQ was used as a HIF (Figure 1). These results suggest that ROS have important roles not only in the conversion of syringic acid to DMBQ, but also in the haustorium formation processes provoked by DMBQ (Figure 9B).

In *Arabidopsis*, it was reported that H_2_O_2_ accumulates at the root tip, especially at the surface of epidermal cells and root hairs in the differentiation zone, and that ${\text{O}}_{2}^{-}$ accumulates in the whole root, and more strongly in the meristem and elongation zone of roots (Dunand et al., 2007). In this study using carboxy-H2DFFDA staining, we did not observe H_2_O_2_ accumulation at the radicle tip of *Striga* before haustorium induction. ${\text{O}}_{2}^{-}$ was detected in the whole plant at low levels and at the radicle tips at high levels, regardless of haustorium formation. In the time-lapse study of H_2_O_2_ accumulation, the root tip swelling signature of early haustorium formation was initiated before H_2_O_2_ accumulation became apparent (Figure 3). Later, the H_2_O_2_ signal was primarily detected in haustorial hairs, similar to that in growing root hairs of normal plants, which agrees with a previous report that haustorial hairs are specialized root hairs in parasitic plants (Cui et al., 2016). Therefore, it is possible that the observed H_2_O_2_ accumulated at later stages of haustorium formation may represent its developmental function on cell growth, that involves cell wall modification and expansion. Previous studies in *Striga asiatica* reported that H_2_O_2_ strongly accumulated at the radicle tip and disappeared about 2 h after HIF treatment (Keyes et al., 2007; Fuller et al., 2017). In contrast to this, we observed almost no H_2_O_2_ fluorescence at the radicle tip before the haustorium induction treatment, with major H_2_O_2_ accumulation appearing after 10–15 h of haustorium induction (Figure 3). It is worth noting that the previous study used *S. asiatica* whereas we used *S. hermonthica*, therefore, the time course of H_2_O_2_ accumulation may be different among different *Striga* species. In addition, we could not detect NO^−^ and ^·^OH in haustoria. Given the characteristic of short half-life of ROS molecules and cell type-dependent permeability of probes, however, further experiments with higher time resolution and different marker combinations are needed to clarify whether various ROS molecules appear at very early stage of haustorium formation.

In our experiments using various ROS inhibitors, the NADPH oxidase inhibitors DPI and PAO strongly inhibited haustorium formation induced by DMBQ and syringic acid. In addition, treatment with a SOD inhibitor, DDC, also inhibited haustorium formation, indicating that H_2_O_2_ production is involved in haustorium formation. However, the effect of DPI could not be restored by addition of external H_2_O_2_, suggesting that *in planta* H_2_O_2_ generation *via* NADPH oxidase activity, which is probably regulated in temporal or spatial manners, is required for haustorium induction. NADPH oxidase enzymes are membrane proteins localized on either the plasma membrane or the chloroplast membrane and are encoded by 10 *Respiratory Burst Oxidase Homolog* (*RBOH*) genes in *Arabidopsis*. *RBOH* genes are expressed in different tissues, among which *RBOH A-C, G*, and *I* are expressed specifically in the root elongation zone (Zimmermann et al., 2004; Sagi and Fluhr, 2006). In *S. asiatica*, Liang et al. (2016) reported that expression of one of the *RBOH* genes *(SaNOX1*) decreased at the early stages (~2 h) of haustorium development accompanied by a H_2_O_2_ reduction at the root tip (Liang et al., 2016). However, it is possible that *S. hermonthica* has several *RBOH* homologs and that each *RBOH* gene has a distinct physiological function. In future, it would be interesting to verify the function of each *RBOH* gene in *S. hermonthica* during haustorium formation in *Striga*. Overall, these results suggest that haustorium induction involves NADPH oxidase-mediated ROS production.

### The Role of Peroxidase in Haustorium Formation

Previous studies proposed that peroxidases are involved in the conversion of syringic acid to DMBQ (Kim et al., 1998). We observed that haustorium-inducing activity was greater with DMBQ treatment than with syringic acid treatment, and that application of HRP increased the haustorium formation rate with syringic acid to a level similar to that of DMBQ. Thus, this result supports the model in which syringic acid is converted to DMBQ by peroxidase. However, HRP also increased the haustorium formation rate with DMBQ treatment. This result suggests that DMBQ may be converted into an unknown compound that has even greater haustorium-induction activity. Alternatively, peroxidase may promote the secretion of another HIF from *Striga*, or raise its sensitivity to HIFs. In our experiments, 10^2^ U/ml HRP induced a haustorium formation rate of 20% without any additional HIFs. The presence of high concentrations of HRP may have caused cell wall degradation in the *Striga* seedlings, releasing small amounts of haustorium-inducing quinones and phenolics. This hypothesis is supported by our previous study in which lignin polymers induced haustorium formation in *S. hermonthica* in the presence of ligninolytic enzymes (Cui et al., 2018). The other possibility is that HRP may directly target the HIF perception signaling pathway, the details of which remain unclear to date. Considering the negative effects of peroxidase inhibitors on haustorium induction, it appears that peroxidases may function to promote haustorium formation.

In conclusion, this study revealed that the haustorium formation rate is affected by the external application of ROS scavengers, NADPH oxidase inhibitors and peroxidase inhibitors, suggesting the importance of endogenous ROS in haustorium formation. Although a previous model indicated the importance of ROS in the production of DMBQ (Figure 9A), our results indicate that ROS, especially H_2_O_2_, are indispensable signals acting downstream of DMBQ. Specifically, a certain level of H_2_O_2_ production by endogenous NADPH oxidase is important in haustorium formation (Figure 9B). In addition, peroxidases may act downstream of DMBQ to enhance haustorium formation signals (Figure 9B). The effects of ROS inhibitors were similar with treatments using DMBQ, syringic acid and rice root extracts, suggesting that these HIFs target the same pathway or have a similar mode of action. Finally, this study provides evidence that ROS have important roles in haustorium formation downstream of the DMBQ signal. In addition, we propose that ROS inhibitors may be useful as the basis for future *Striga* control reagents.

## Data Availability

All datasets generated for this study are included in the manuscript and/or the Supplementary Files.

## Author Contributions

SW, SC, and SY conceived the idea of this study, designed the experiments and wrote the manuscript. SW and SC performed the experiments and data analysis.

### Conflict of Interest Statement

The authors declare that the research was conducted in the absence of any commercial or financial relationships that could be construed as a potential conflict of interest.

## Supplementary Material

Supplementary Figure S1 Visualization of NO^−^ and ^·^OH. For visualization of NO^−^ **(A–C)** and ^·^OH **(D–F)**, haustoria of *S. hermonthica* induced by 10 μM DMBQ **(B,E)** or syringic acid **(C,F)** for 24 h were stained with 10 μM DAF-2 DA **(A–C)** or 10 μM APF **(D–F)** for 30 min. Radicals grown in water **(A, D)** were observed as a control. Left and right panels show confocal microscope images and DIC images, respectively. The scale bar indicates 500 μm.

Supplementary Figure S2 Images of *S. hermonthica* seedlings treated with ROS inhibitors and DMBQ or syringic acid. Germinated *S. hermonthica* seedlings were exposed to each inhibitor at indicated concentrations with or without (control) 10 μM DMBQ or syringic acid and observed after 24 h of treatment. White arrowheads indicate brownish radicle tip by high concentration of esculetin treatment.

Supplementary Figure S3 Effects of SHAM and PAO on haustorium formation. **(A)** Concentration-dependent inhibition of SHAM on haustorium formation induced by DMBQ or syringic acid. **(B)** Effect of PAO on haustorium induction by DMBQ or syringic acid. Error bars indicate SE (*n* = 3), and NT, not tested. Different lower-case letters represent significant differences as determined by a Tukey HSD test (*p* < 0.05).

Supplementary Figure S4 Effects of ROS inhibitors on haustorium formation induced by rice root extracts. Haustorium formation rates were calculated after *S. hermonthica* seedlings were treated with 0.5% root extracts from cultivars Nipponbare and Koshihikari in the absence or presence of various ROS inhibitors. The concentration of each chemical is shown at the top left of each graph. Error bars indicate SE (*n* = 3), and NT, not tested. Different lower-case letters represent significant differences as determined by a Tukey HSD test (*p* < 0.05).

Supplementary Table S1 Chemicals used in this study.

Supplementary Movie S1 Time-lapse movie of H_2_O_2_ accumulations during haustorium formation. Time-lapse images H_2_O_2_ accumulation visualized by carboxy-H2DFFDA staining were taken every 30 min after with **(A)** or without **(B)** DMBQ treatment for 24 h.
