# Supplementary material for: Reactive Oxygen Species (ROS) Generation Is Indispensable for Haustorium Formation of the Root Parasitic Plant Striga hermonthica
Source: Front Plant Sci. 2019 Mar 22;10:328. doi: 10.3389/fpls.2019.00328 (PMC6438919; doi:10.3389/fpls.2019.00328)
Supplement: Supplementary Figure S1 — Visualization of NO− and ·OH. For visualization of NO− (A–C) and ·OH (D–F), haustoria of S. hermonthica induced by 10 μM DMBQ (B,E) or syringic acid (C,F) for 24 h were stained with 10 μM DAF-2 DA (A–C) or 10 μM APF (D–F) for 30 min. Radicals grown in water (A, D) were observed as a control. Left and right panels show confocal microscope images and DIC images, respectively. The scale bar indicates 500 μm. [file Data_Sheet_1.pdf]

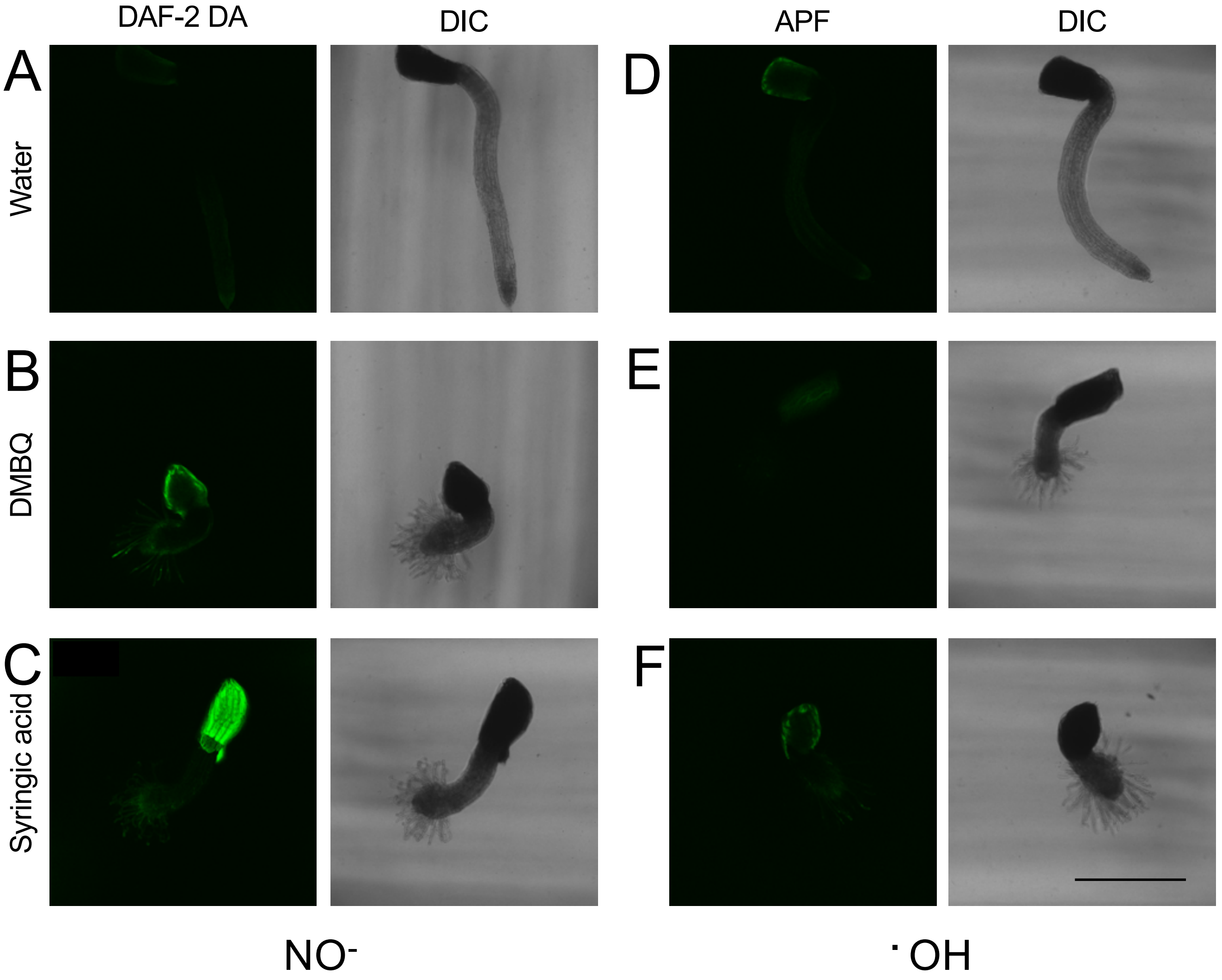

Supplementary Figure S1 Visualization of NO^- and $^{\cdot}OH$

For visualization of NO^- (A-C) and $^{\cdot}OH$ (D-F), haustoria of *S. hermonthica* induced by 10 μM DMBQ (B and E) or syringic acid (C and F) for 24 h were stained with 10 μM DAF-2 DA (A-C) or 10 μM APF (D-F) for 30 min. Radicals grown in water (A, D) were observed as a control. Left and right panels show confocal microscope images and DIC images, respectively. The scale bar indicates 500 μm.

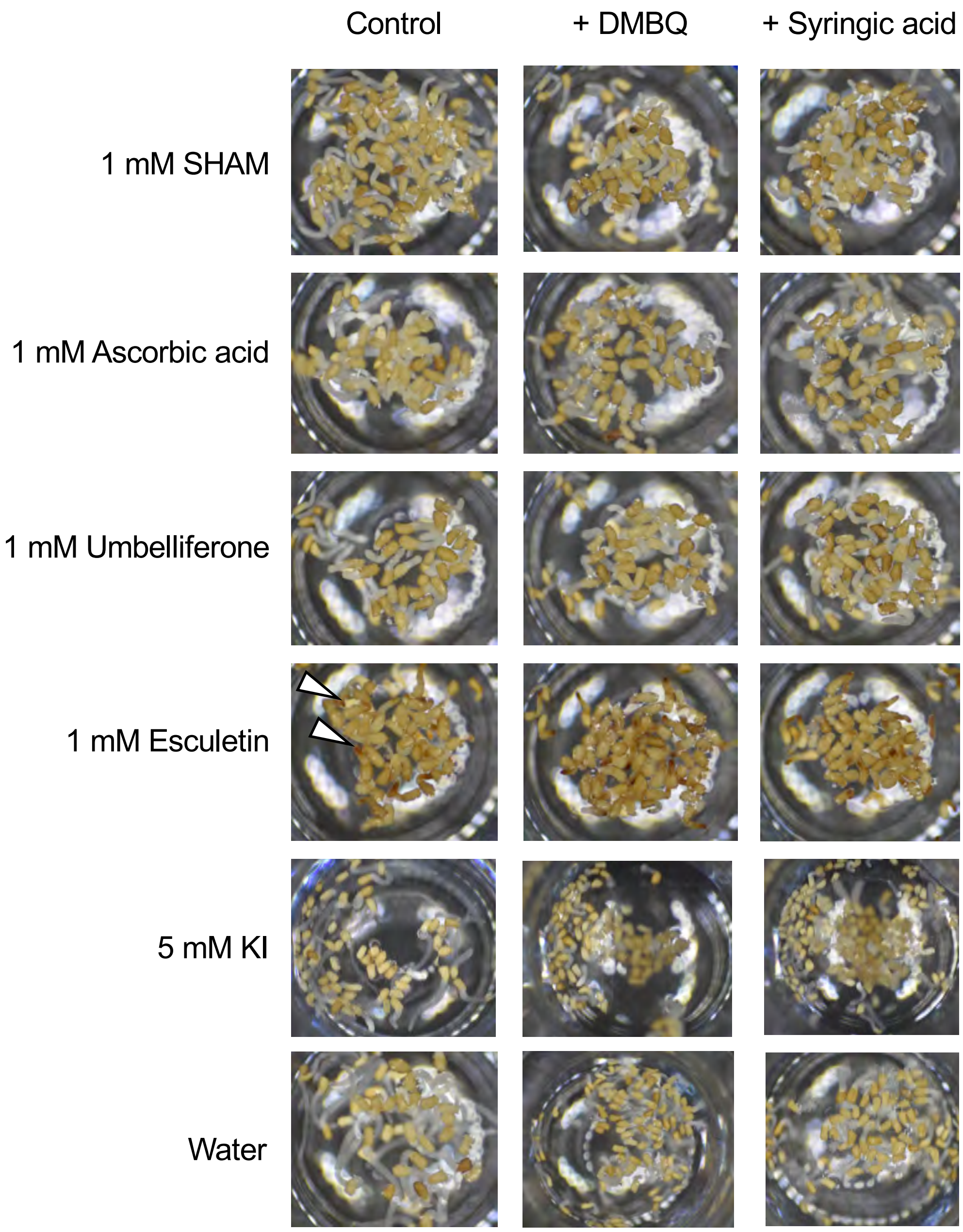

Supplementary Figure S2 Images of *S. hermonthica* seedlings treated with ROS inhibitors and DMBQ or syringic acid

Germinated *S. hermonthica* seedlings were exposed to each inhibitor at indicated concentrations with or without (control) 10 µM DMBQ or syringic acid and observed after 24 h of treatment. White arrowheads indicate brownish radicle tip by high concentration of esculetin treatment.

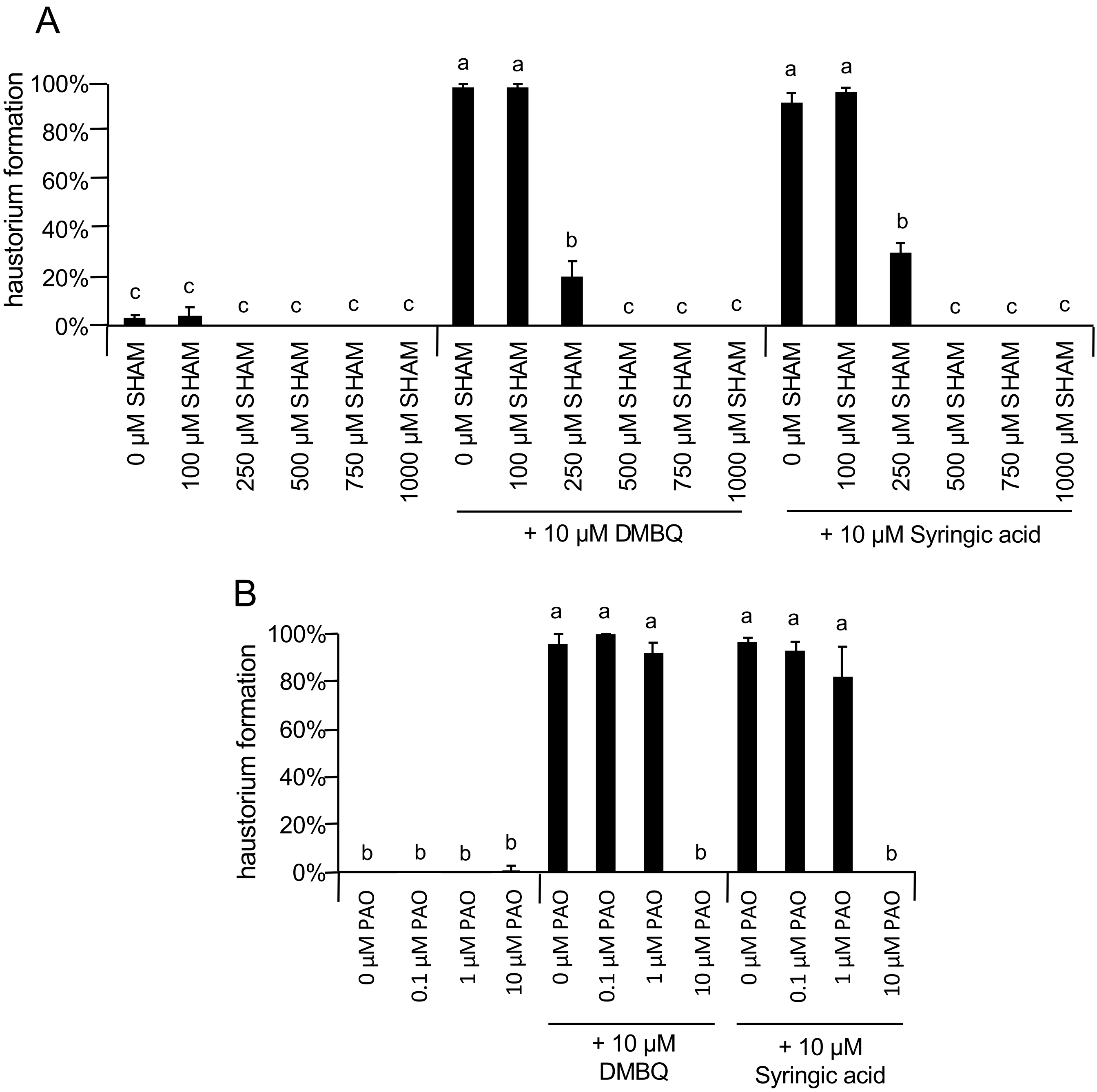

Supplementary Figure S3 Effects of SHAM and PAO on haustorium formation

(A) Concentration-dependent inhibition of SHAM on haustorium formation induced by DMBQ or syringic acid. (B) Effect of PAO on haustorium induction by DMBQ or syringic acid. Error bars indicate SE ($n = 3$), and NT = not tested. Different lower-case letters represent significant differences as determined by a Tukey HSD test ($p < 0.05$).

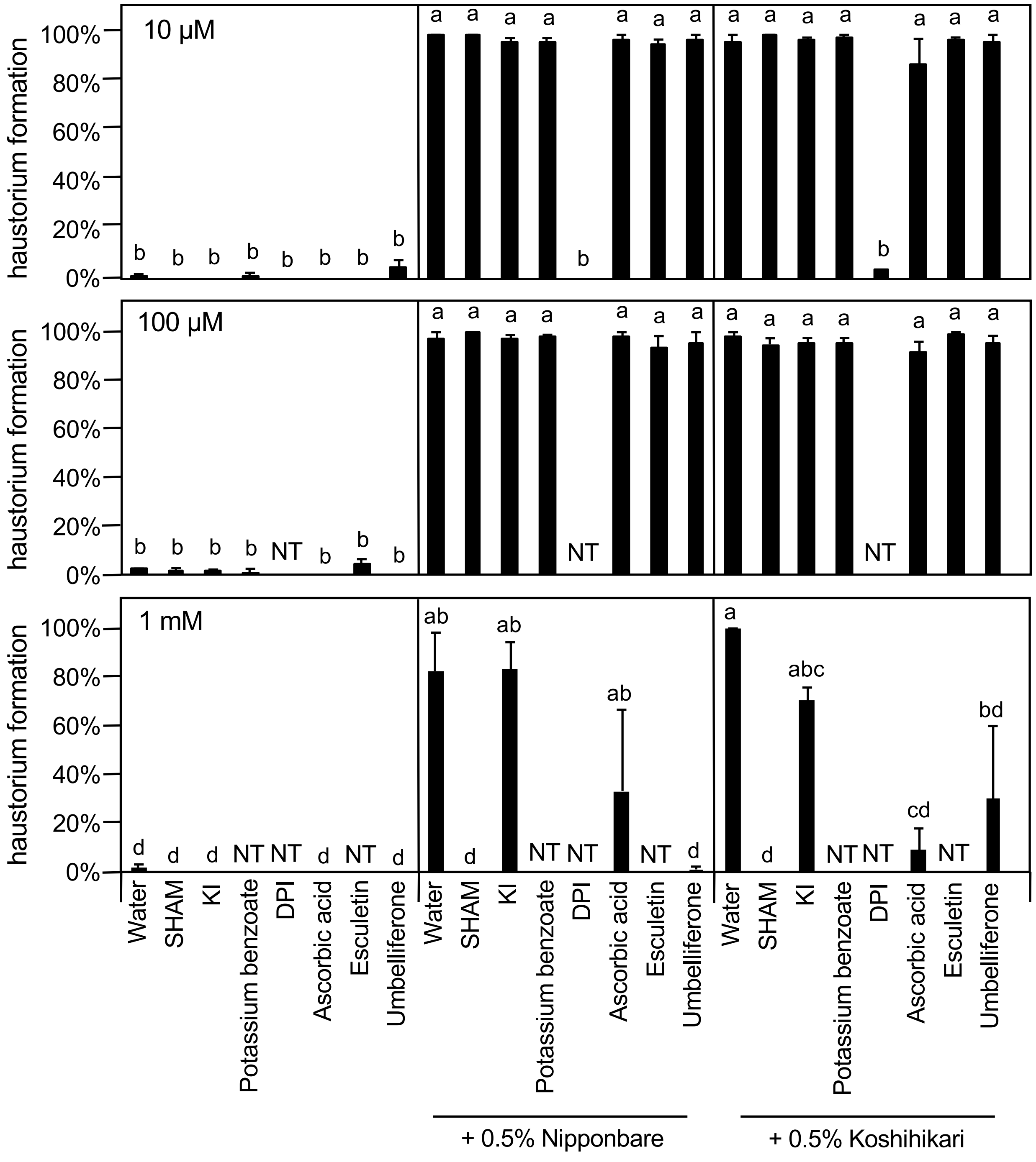

Supplementary Figure S4 Effects of ROS inhibitors on haustorium formation induced by rice root extracts

Haustorium formation rates were calculated after *S. hermonthica* seedlings were treated with 0.5 % root extracts from cultivars Nipponbare and Koshihikari in the absence or presence of various ROS inhibitors. The concentration of each chemical is shown at the top left of each graph. Error bars indicate SE (n = 3), and NT = not tested. Different lower-case letters represent significant differences as determined by a Tukey HSD test ($p < 0.05$).

Supplementary Table S1. Chemicals used in this study

Chemical name	Company	Stock concentration	Role
Strigol	-	10 µM in acetone	Induction of germination
2,6-dimethoxy-1,4-benzoquinone (DMBQ)	Sigma-Aldrich	10 mM in DMSO	Haustorium inducing factor
Syringic acid	Sigma-Aldrich	10 mM in DMSO	Haustorium inducing factor
Salicylhydroxamic acid (SHAM)	Tokyo Chemical Industry Co., Ltd	10 mM in DMSO	Peroxidase inhibtor
		100 mM in DMSO	
Potassium iodide (KI)	Wako Pure Chemical Industries, Ltd	10 mM in DMSO	Hydrogen peroxide scavenger
		100 mM in DMSO	
		100 mM in water	
Potassium benzoate	Wako Pure Chemical Industries, Ltd	10 mM in DMSO	Scavenger of hydroxyl radicals
Diphenyleneiodonium (DPI)	Funakoshi Frontiers Life Science	10 mM in DMSO	NADPH oxidase inhibitor
L-ascorbic acid	Wako Pure Chemical Industries, Ltd	10 mM in DMSO	Scavenger of radical species
		100 mM in DMSO	
Umbelliferone	Tokyo Chemical Industry Co., Ltd	10 mM in DMSO	Peroxidase modulator
		100 mM in DMSO	
6,7-dihydroxycoumarin (Esculetin)	Sigma-Aldrich	10 mM in DMSO	Peroxidase modulator
Phenylarsine oxide (PAO)	Sigma-Aldrich	10 mM in DMSO	NADPH oxidase inhibitor
Diethyldithiocarbamate trihydrate (DDC)	Sigma-Aldrich	10 mM in DMSO	SOD inhibitor
